# Profile of Microbial Keratitis after Corneal Collagen Cross-Linking

**DOI:** 10.1155/2014/340509

**Published:** 2014-09-11

**Authors:** Rohit Shetty, Luci Kaweri, Rudy M. M. A. Nuijts, Harsha Nagaraja, Vishal Arora, Rajesh S. Kumar

**Affiliations:** ^1^Narayana Nethralaya Eye Hospital Bangalore, Narayana Nethralaya 121/C, Chord Road, 1st “R” Block, Rajajinagar, Bangalore, Karnataka 560 010, India; ^2^Department of Ophthalmology, University Hospital Maastricht, P. Debyelaan 25, 6229 HX, Maastricht, The Netherlands

## Abstract

*Purpose*. To report the profile of microbial keratitis occurring after corneal collagen cross-linking (CXL) in keratoconus patients. *Methods*. A retrospective analysis of 2350 patients (1715 conventional CXL, 310 transepithelial CXL, and 325 accelerated CXL) over 7 years (from January 2007 to January 2014) of progressive keratoconus, who underwent CXL at a tertiary eye care centre, was performed. Clinical findings, treatment, and course of disease of four eyes that developed postprocedural moxifloxacin resistant *Staphylococcus aureus* (MXRSA) infectious keratitis are highlighted. *Results*. Four eyes that underwent CXL (0.0017%) had corneal infiltrates. All eyes that developed keratitis had conventional CXL. Corneal infiltrates were noted on the third postoperative day. Gram's stain as well as culture reported MXRSA as the causative agent in all cases. Polymerase chain reaction (PCR) in each case was positive for eubacterial genome. All patients were treated with fortified antibiotic eye drops, following which keratitis resolved over a 6-week period with scarring. All these patients were on long-term preoperative oral/topical steroids for chronic disorders (chronic vernal keratoconjunctivitis, bronchial asthma, and chronic eczema). *Conclusion*. The incidence of infectious keratitis after CXL is a rare complication (0.0017%). MXRSA is a potential organism for causing post-CXL keratitis and should be identified early and treated aggressively with fortified antibiotics.

## 1. Introduction

The treatment of keratoconus has been revolutionized with the introduction of newer treatment modalities like corneal collagen cross-linking. Corneal collagen cross-linking with riboflavin (CXL) has been reported to increase the mechanical rigidity of cornea and thus delay or even halt the progression of keratoconus [1, 2]. CXL is a relatively safe surgery without sight-threatening complications [1]. It does not alter the ocular surface as evidenced by no significant changes in objective dry eye parameters after CXL [3]. Its long term stability, safety, and efficacy coupled with other procedures have been reported [4, 5]. Customization of CXL is in vogue [6]. However, microbial keratitis due to varied etiology including herpetic, bacterial (*Escherichia coli*,* Pseudomonas*,* Staphylococcus* and* Streptococcus*), and* Acanthamoeba* has been reported after CXL [7–12]. We have evaluated the incidence of post-CXL infectious keratitis among keratoconus patients in our centre and reported a series of patients who developed moxifloxacin resistant* Staphylococcus aureus* (MXRSA) keratitis following CXL.

## 2. Materials and Methods

In this retrospective analysis, records of 2350 patients undergoing CXL (1715 conventional CXL, 310 transepithelial CXL, and 325 accelerated CXL) in the last 7 years at a tertiary eye care centre in southern India were reviewed. All patients were diagnosed with progressive keratoconus (an increase of 0.5 diopter (D) or more in two or more keratometric values in the steep meridian between two sagittal curve maps or a decrease in corneal thickness of 10% or more at the thinnest point between two pachymetry maps on Pentacam (Oculus, Wetzlar, Germany) in the preceding six months) and required surgical intervention [13]. Protocol of CXL used was based on the thinnest pachymetry values. Those with thinnest pachymetry more than 450 microns underwent conventional or accelerated CXL. Patients with pachymetry less than 450 microns were chosen for tranepithelial CXL.

All four patients who developed postprocedure keratitis underwent conventional CXL at our hospital on different days. CXL being a safe procedure, sending conjunctival swab preoperatively, is not routinely practised. Exceptions to this are one eyed patients. Prophylactic antibiotics, moxifloxacin hydrochloride 0.5% (Vigamox, Alcon, USA) three times a day, were started 3 days prior to the surgery. Conventional CXL was performed using the standard protocol advised by Spoerl et al. [14]. Local anaesthesia consisting of proparacaine hydrochloride 0.5% (Paracain, Sunways Pvt. Ltd., India) eye drop was instilled in the treated eye under aseptic conditions. A lid speculum was placed in the fornix. The central 8.0 mm of the corneal epithelium was debrided using an epithelial scraper. Thirty minutes prior to the actual irradiation, 1 drop of riboflavin 0.1% photosensitizer solution containing 10 mg riboflavin-5-phosphate (in 10 mL 20% wt/vol dextran 500) was instilled onto the debrided central cornea every 5 minutes. The corneas were then irradiated with UVA for 30 minutes (irradiance 3 mW/cm^2^; dose 5.4 J/cm^2^) using a 370 nm UVA double-diode light source. Following CXL a BCL (bandage contact lens, Ciba Vision, CIBA Vision Corp, Duluth, GA) was inserted; the BCL was removed by the surgeon once the epithelial defect had healed. Transepithelial CXL involved soaking of nondebrided corneas with 0.25% riboflavin every 2 minutes for a period of 30 minutes. This was followed by irradiation for a period of 2 minutes 40 seconds (irradiance 45 mW/cm^2^; dose 7.2 J/cm^2^). In accelerated CXL, corneal epithelium was debrided either manually using a scraper or with an excimer laser (phototherapeutic keratectomy). After a soaking time of twenty minutes with riboflavin 0.1%, an irradiation of 5.4 J/cm^2^ (30 mW/cm^2^ for 3 minutes; 18 mW/cm^2^ for 5 minutes; and 9 mW/cm^2^ for 10 minutes) was delivered. Postoperative treatment for all patients included topical antibiotics, moxifloxacin hydrochloride 0.5%, 3 times a day for 1 week. We did not start steroids in the immediate postoperative period as our protocol is to defer steroids till the epithelial defect has healed completely. As per protocol, all patients were followed up on days 1 and 3, and then at 1 week, 1 month, 3 months, and 6 months after procedure.

## 3. Results

**Figure 1:**
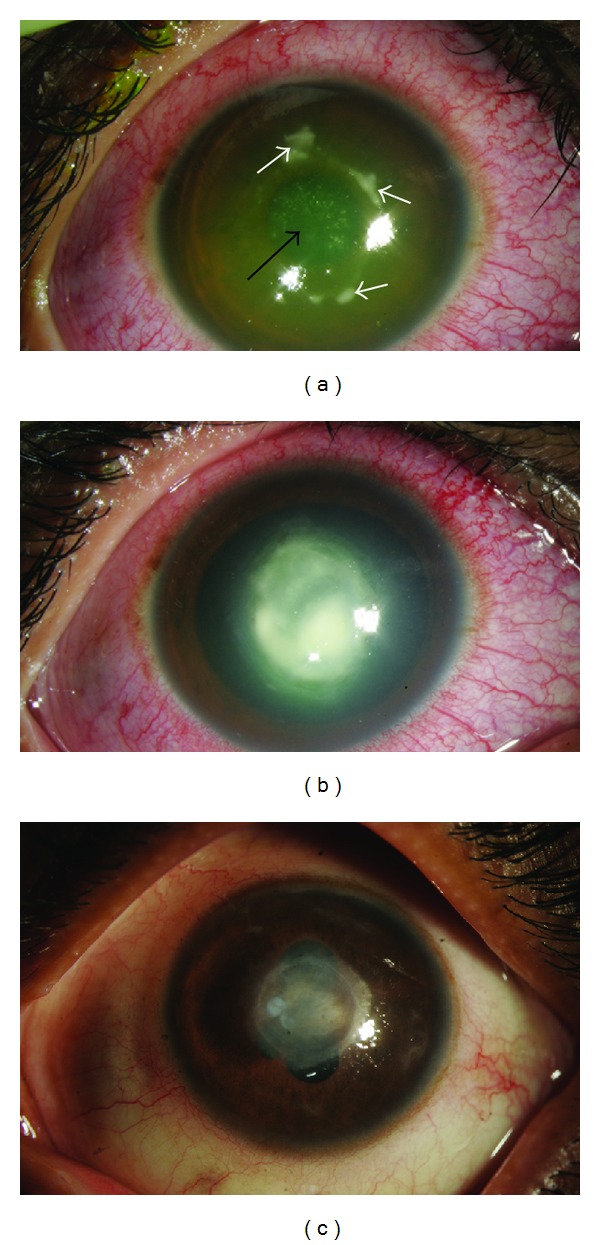
Slit lamp photographs showing multifocal anterior stromal infiltrates (arrows) with overlying epithelial defect (3rd postoperative day) (a), coalescence of the infiltrates (6th postoperative day) (b), and anterior stromal scars with no active infiltrate (four weeks after procedure) (c).

There were no intraoperative complications with any of the patients. Patients were seen on the third postoperative day for BCL removal. All 4 patients reported here had complaints of severe pain, watering, and photophobia.  Table 1 highlights the clinical profile of these patients. On examination, the lids were edematous and conjunctiva showed diffuse congestion. There were multifocal anterior stromal infiltrates with well circumscribed margins on the cornea associated with edema around the infiltrates in the central 4 mm and an overlying epithelial defect; the anterior chamber was quiet (Figure 1(a)); BCL was present in situ. A complete microbiological workup was ordered for all cases; the BCL was also sent for culture. Gram's stain showed gram positive cocci, while potassium hydroxide (KOH) wet mount did not show any fungi. All patients were advised to use moxifloxacin eye drops hourly till culture and sensitivity test reports were received; they were reviewed daily. Steroids were not started in these cases. All eyes showed an increase in both symptoms (worsening of pain and photophobia) and signs (increase in the infiltrate size and coalescing) over the next couple of days with an active anterior chamber reaction (Figure 1(b)). Polymerase chain reaction (PCR) was positive for eubacterial genome. The bacterial culture (including the one from the BCL) showed significant number of* Staphylococcus aureus* which on antibiogram were resistant to moxifloxacin while being sensitive to gatifloxacin, tobramycin, and cefazoline. Topical medications were changed immediately to hourly fortified cefazoline 5% and tobramycin 1.3%. Patients were reviewed daily for 3 days and then on alternate days. Over the next week they showed a decrease in infiltrate size. After six weeks none of the patients had any active infiltrate; only anterior stromal scars were visible (Figure 1(c)). Fluorometholone acetonide 0.1% (FML, Allergan Ltd.) was added to reduce the scarring and tapered over three weeks. None of the patients had any vitritis during the entire course; all patients categorically stated that they had not handled their contact lens after insertion by the ophthalmologist.

**Figure 2:**
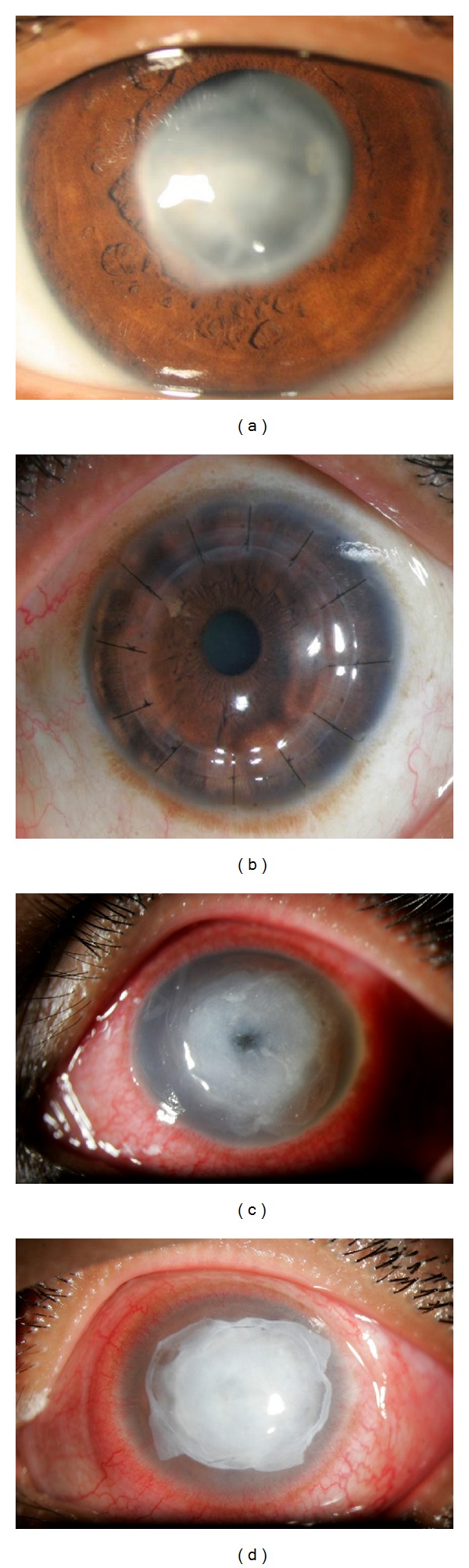
Slit lamp photographs of patient 1 who had dense stromal scarring (a) 2 months after resolution of keratitis; the patient underwent femtosecond enabled keratoplasty (FEK) (b); patient 4 developed stromal melt a week after resolution of keratitis (c) and underwent successful amniotic membrane grafting (d).

Two months after resolution of keratitis, patient 1 underwent femtosecond enabled full thickness penetrating keratoplasty with good clinical outcome (Figures 2(a) and 2(b)) (Table 1). Patient 2 was given rigid gas permeable lenses. Patient 3 has been advised to undergo penetrating keratoplasty. Since patient 4 had stromal melt, amniotic membrane grafting was done, which stopped further lysis and healed with scarring (Figures 2(c) and 2(d)) (Table 1).

## 4. Discussion

Cross-linking is currently one of the most widely used treatment strategies for keratoconus. Despite the well-established safety profile of the procedure, there have been reports with regard to postoperative infections following CXL with riboflavin and UVA (Table 2). Kymionis et al. described a patient who developed epithelial herpetic keratitis and iritis after CXL treatment and hypothesised that UVA light could be a potent stimulus to induce reactivation of latent HSV infections even in patients with no history of clinical herpes virus ocular infections [7]. They also postulated that corneal epithelial/stromal trauma or actual damage of the corneal nerves could be the mechanism of HSV reactivation and also the use of topical corticosteroids may be additional risk factors. Another group reported a case of post-CXL corneal melt wherein corneal scrapping was positive for* Acanthamoeba*; the patient had to undergo therapeutic keratoplasty; their patient washed eyelids and face with tap water with the BCL in situ which was a potential risk factor [9]. Zamora and Males reported that their patient, who presented with culture proven polymicrobial keratitis 3 days following CXL, had a history of handling the BCL in the immediate postoperative period; they postulated that this could have been a risk factor for keratitis [11]. Though none of the patients claimed to have handled the BCL in our series, it is difficult to ascertain whether the use of BCL is in itself a potential risk factor for the development of keratitis post-CXL.

The original treatment protocol (Wollensak et al.) proposed the use of antibiotic ointments in the postoperative period after CXL [1]. Various other studies have highlighted the use of postoperative steroids and/or nonsteroidal anti-inflammatory drugs (NSAIDs) along with an antibiotic agent [7, 8, 10]. However, it is also known that the use of topical corticosteroids and/or NSAIDs has the potential to exacerbate an infection [15]. Hence, in our practice, we do not use topical steroids till the epithelium has healed. In the reported cases, steroids were not started as infection was noticed on the third postoperative day. We have also modified our protocol to follow up our patients every day till the epithelium is healed completely. We also recommend the use of BCL after procedure as it enhances epithelial healing and decreases discomfort [16].

Unlike other drugs like ofloxacin [17], voriconazole [17], pilocarpine [18] and fluorescein [19] which have reduced penetration through cross-linked cornea, penetration of moxifloxacin into the anterior chamber has been proven to be unaltered by CXL [20]. Moxifloxacin has enhanced potency against* S. aureus* and higher bactericidal activity against highly resistant strains than ciprofloxacin [21]. When keratitis is noted, the accepted practice is to perform a culture and sensitivity tests to determine the appropriate antibiotic that would be ideal for the organism and then change the treatment regimen. In our series, since Gram's stain showed* S. aureus* as the causative organism and MXRSA keratitis has not been reported to date, we did not change the drug, but rather increased the frequency.

Moxifloxacin, a topical fourth generation quinolone is four- to eight-fold more potent against* S. aureus* than ciprofloxacin, despite similar inhibition of topoisomerase IV and DNA gyrase by both the drugs. Moxifloxacin, similar to other 8-methoxyquinolones, has been shown to preferentially target topoisomerase IV in vivo in* S. aureus* [21]. Thus, even a single mutation in topoisomerase IV could contribute to moxifloxacin resistance. It might be possible that mutations may have been caused in the* S. aureus* species in our series due to the UVA radiation used during CXL, similar to events like activation of latent herpes virus [7]. We understand that this is currently a hypothesis at best; a more detailed research and understanding of the mechanisms involved is needed.

Our group has already reported the effectiveness of CXL in treating nonresolving microbial keratitis with superficial stromal involvement [22]. However it is interesting that CXL itself might be a precipitating factor in causing keratitis. The common link between all four patients in our series could potentially have been the long-term use of preoperative steroids (topical/systemic) (Table 1). This might have led to an immunocompromised status. Previous studies have shown that there are changes in ocular flora due to the chronic use of topical steroids in keratoconus patients with VKC. This could possibly lead to an increased risk of postoperative keratitis [23]. Hence it might be important to monitor the use of topical/oral steroids in these patients and inform the patient and treating physician of the potential risks.

Both epithelial debridement and CXL have been shown to cause damage to stromal keratocytes which have a role in corneal immune response [24–26]. This might suggest that all high risk patients like those on oral or topical steroids or other immunosuppressive drugs should be counselled prior to surgery and followed up more carefully. Simultaneous bilateral surgeries should be avoided in such patients. Drug resistant infections should also be kept in mind. It is interesting to note that in all the reported cases published and also in our centre, the infections occurred after conventional CXL. There are no reports of infections after accelerated (KXL) or transepithelial cross-linking (TEKXL). We could hypothesize that the longer exposure to UV-A and duration of the conventional CXL might be a potential precipitating factor. There might be a role for TEKXL or KXL as an alternative to CXL, as the total procedure time is significantly lesser in these procedures; further studies to validate this are needed.

Moxifloxacin is preferred by most surgeons for surgical prophylaxis [27]. It has proven advantageous over older fluoroquinolones as well as other topically available antimicrobials, has a broader spectrum of action and excellent penetration into eye tissues, and is able to deliver a concentration thousands of times the minimum inhibitory concentration [28–31]. To the best of our knowledge, this is the first report of MXRSA keratitis following CXL. Culture and PCR allowed timely intervention. If keratitis develops in spite of antibiotic coverage, a high level of suspicion of drug resistance should be present. While treating postprocedure keratitis, the proper course of action might be to use alternate or fortified antibiotics rather than increasing the frequency of the fluoroquinolones in the interim.

The infection might reduce, but the visual morbidity may still be high. A larger multicentric cohort is needed to validate these conclusions.

## Figures and Tables

**Table 1:** Clinical profile of patients who developed keratitis after collagen cross-linking.

Clinical profile (Age/Sex)	Associated conditions and treatment for the same duration		Preoperative BCVA	Procedure	Day of presentation of symptoms from day of surgery	Treatment	Time for resolution of symptoms	Rehabilitation	Final BCVA
Patient 1 (27/F)	Bronchial asthma (12 years)	Oral/Inhalational steroids (12 years)	20/30	Conventional CXL	3	Fortified antibiotics	6 weeks	FEK	20/20
Patient 2 (18/M)	Vernal catarrh (10 years)	Topical steroids (10 years)	20/20	Conventional CXL	3	Fortified antibiotics	4 weeks	RGP	20/30
Patient 3 (25/M)	Eczema (5 years)	Oral cyclophosphamide (5 years)	20/20	Conventional CXL	3	Fortified antibiotics	5 weeks	Advised PKP	20/120
Patient 4 (16/M)	Vernal catarrh (6 years)	Topical steroids (6 years)	20/20	Conventional CXL	3	Fortified antibiotics	5 weeks	AMG, under follow-up	20/200

M: male, F: female, BCVA: best corrected visual acuity, FEK: femtosecond enabled penetrating keratoplasty, PKP: penetrating keratoplasty, RGP: rigid gas permeable lens, and AMG: amniotic membrane grafting.

**Table 2:** Reports of keratitis after collagen cross-linking in literature.

Author	Number of cases reported	CDVA	Time to presentation (Days)	Organisms	Final CDVA
Pollhammer and Cursiefen [8]	1	20/400	3	*Escherichia coli *	20/63
Rama et al. [9]	1	—	5	*Acanthamoeba *	20/40
Pérez-Santonja et al. [10]	1	20/20	2	*Staphylococcus epidermidis *	20/22
Zamora and Males [11]	1	CF	3	*Streptococcus salivarius*,* Streptococcus oralis*, and coagulase-negative *Staphylococcus* species	20/50
Sharma et al. [12]	1	HMNF	3	*Pseudomonas aeruginosa *	20/200

CF: counting fingers, HMNF: hand movements near to face, and CDVA: corrected distance visual acuity.
